# Racial differences in prevalence and anatomical distribution of tarsal coalition

**DOI:** 10.1038/s41598-022-26049-6

**Published:** 2022-12-13

**Authors:** Jeong Jin Park, Hyun Gyu Seok, In Ha Woo, Chul Hyun Park

**Affiliations:** 1 Department of Orthopaedic Surgery, Yeungnam University Hospital, Yeungnam University Medical Center, 170 Hyeonchung-ro, Nam-gu, Daegu, 42415 South Korea; 2 Department of Orthopaedic Surgery, Yeungnam University Medical Center, Yeungnam University College of Medicine, 170 Hyeonchung-ro, Nam-gu, Daegu, 42415 South Korea

**Keywords:** Anatomy, Diseases

## Abstract

Previous studies have reported a prevalence of tarsal coalition of 0.03–13%. Calcaneonavicular coalition is known as main anatomical type, and the bilateral occurrence of tarsal coalition is known to be 50% or more. These are the results of studies on Caucasians, there have been few studies targeting large number of East Asians so far. We hypothesized that the prevalence and characteristics of tarsal coalition in East Asians might differ from those in Caucasians. The medical records of 839 patients who underwent bilateral computed tomography on foot and ankle in our hospital from January 2012 to April 2021 were retrospectively reviewed. The overall prevalence was 6.0%, talocalcaneal coalition was the most common anatomical type. The overall bilateral occurrence was 56.5%, talocalcaneal coalition had the highest bilateral occurrence (76.0%) among anatomical types. Isolated union of the posterior facet was the most common subtype of talocalcaneal coalition (43.2%). Talocalcaneal coalition had a significantly higher proportion of coalition-related symptomatic patients than calcaneonavicular coalition (*p* = 0.019). Our study showed a similar trend to other East Asian studies, confirming the existence of racial differences. The possibility of tarsal coalition in foot and ankle patients in East Asians should always be considered, and bilateral examination is essential for diagnosis.

## Introduction

Tarsal coalition is as state in which two or more different tarsal bones are united, and maybe fibrous, cartilaginous, or osseous^1–3^. Failure of mesenchymal separation has been suggested to be the primary cause of tarsal coalition, which is presumed to be due to an autosomal dominant inheritance pattern with high penetrance^1–5^. Tarsal coalition is associated with diminished motion of affected joints and often leads to pathologic conditions including rigid flat foot, peroneal spasm, and secondary degenerative change on adjacent joint^3,6–10^. Therefore, understanding the prevalence and characteristics of tarsal coalition has clinically important value.

Previous studies have reported a prevalence of tarsal coalition of 0.03–1%^11–14^. Harris et al.^11^ evaluated the prevalence of peroneal spastic flatfoot using physical examination. Other authors assessed tarsal coalition by plain radiography in patients with painful feet or primary foot diseases. Considering that most of the tarsal coalition patients are asymptomatic, and plain radiography is not suitable for diagnosing non-osseous coalitions, the prevalence of tarsal coalitions is expected to be much higher. Recent studies using cadavers or magnetic resonance imaging (MRI) have reported prevalence of 11.5–13% in Caucasians^15,16^.

The most common anatomical type of tarsal coalition is known as calcaneonavicular (CN) coalition^5,15,17^. Bilateral occurrence have been reported in 50–68% of cases^2,5,18–20^, and multiple tarsal coalition are very rare^21–23^. Talocalcaneal (TC) coalition is further subdivided according to the location of union in the subtalar joint, and middle facet is known as the most common coalition site^19,24–27^. But, most of these results were obtained in Caucasian studies. According to some East Asian studies which used computed tomography (CT) or MRI for diagnosis, TC coalition has a higher proportion than CN coalition, and TC coalition most commonly involves the posterior subtalar facet^28–32^. It appears that the anatomical distributions of tarsal coalition might differ between races.

Most East Asian studies were conducted on patients diagnosed with tarsal coalition^30,32,33^. In addition, Kim et al.^31^ conducted a study on a large number of 4711 patients, but it was insufficient to have representativeness of the population as soldiers with ankle sprains and fractures were the subjects of the study. Therefore, East Asian studies on tarsal coalition are still insufficient to evaluate prevalence and anatomical distribution and to support the possibility of racial difference. And there is no study assessed bilaterality in a large sample to the best of our knowledge.

This study was performed using both-side CT in a large Korean cohort of 839 patients. We evaluated the prevalence, anatomical distributions, and bilateral occurrence of tarsal coalition. In addition, we assessed the proportion of symptomatic patients associated with tarsal coalition. Racial differences and clinical significance will be discussed based on the results of this study and the trends of previous studies. We hypothesized that the characteristics of tarsal coalition in East Asians might differ from those previously reported in Caucasians.

## Materials and methods

This study was reviewed and approved beforehand by the Institutional Review Board (IRB) of Yeungnam Medical Center (IRB number: 2021-10-029), which waived the requirement for informed consent because of the retrospective design of the study. All research processes were conducted in accordance with the appropriate regulations and guidelines, and this study was performed in accordance with the provisions of the Declaration of Helsinki. The medical records of all patients who underwent bilateral CT at our hospital from January 2012 to April 2021 were retrospectively reviewed, and 1028 patients were identified. Patients with abnormal anatomical structures or with the possibility of secondary tarsal coalition were excluded from the study (Table 1). Finally, this study was conducted on 839 patients (492 men and 347 women) of average age 44 years (range, 13–75 years).

**Table 1:** Exclusion list.

Exclusion criteria	Excluded patients (n = 189)
Fracture around foot and ankle	135
Severe osteoarthritic change on subtalar joint	31
Tumor	10
Infection	10
Congenital anomalies of lower extremity	3

Values are presented as number.

All patients were evaluated for the presence and location of the tarsal coalition using coronal, sagittal, transverse, and three-dimensional reconstruction images of bilateral CTs. CT was used as a main imaging technique for identification of tarsal coalition because it is advantageous in understanding the complex anatomical structure of tarsal bone^34,35^. All CTs were taken in the same facility using the same protocol. Images were obtained using a SOMATOM definition AS+ unit (Siemens, Germany) using a bone algorithm. Typical scan parameters were as follows; field of view, 25 cm; peak voltage, 120 kVp; quality reference, 82 mAs; scan time per slice, 1 s; and slice thickness, 2 mm. All radiographic evaluations were performed using a picture archiving and communication system (Maroview1, version 5.4; Marotech, Seoul, Korea) in Digital Imaging and Communicating in Medicine (DICOM) format.

The diagnostic criteria of tarsal coalition used in this study were as follows: the presence of a bone bridge, narrowing of the joint surface, irregular cortical bone surface, subchondral bone sclerosis, and cyst formation^36^. The overall prevalence of tarsal coalitions was evaluated as the proportion of patients who displayed these features on CT images among the patients included in this study. Proportions of tarsal coalition patients by groups according to age and gender were also evaluated and ages were classified using 20-year intervals.

**Figure 1:**
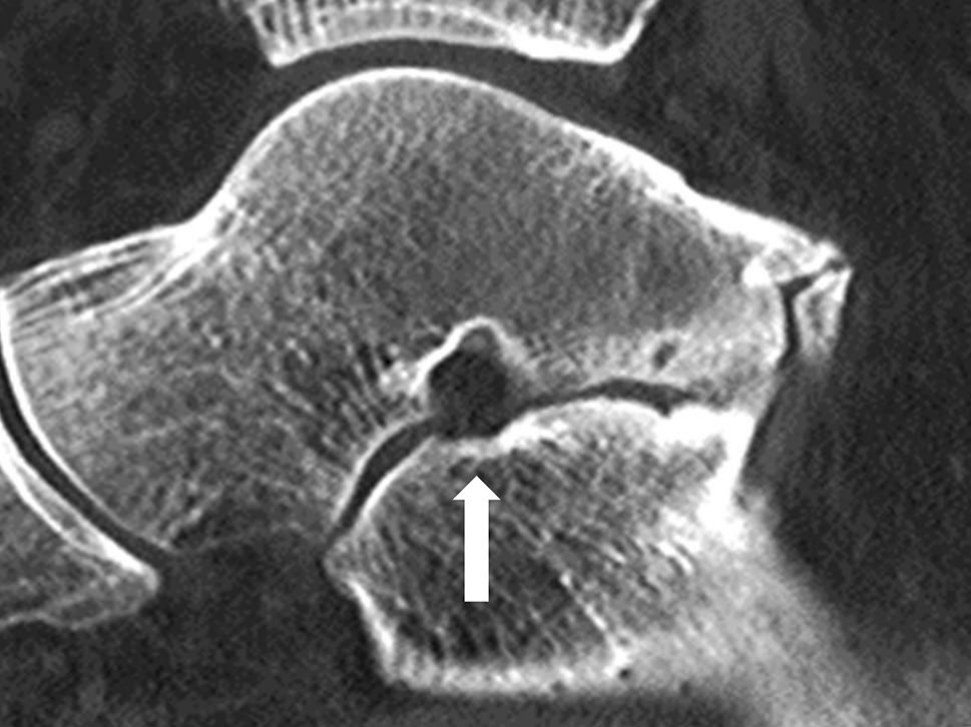
The canalis-tarsi between the middle and posterior subtalar facets.

**Figure 2:**
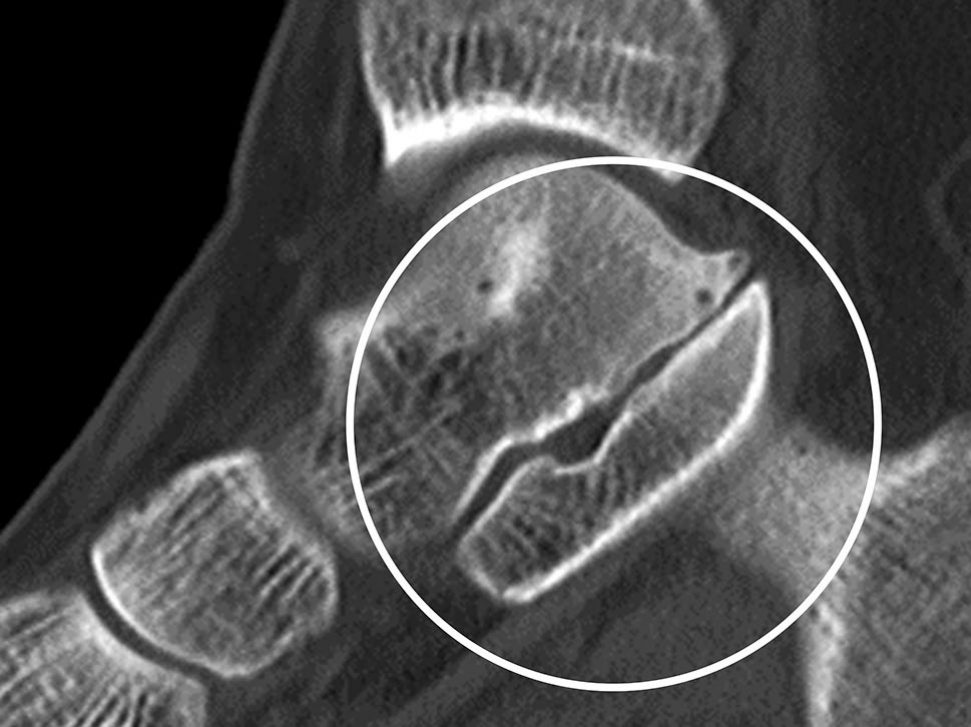
Computed tomography findings of non-osseous talocalcaneal coalition which is similar to degenerative osteoarthritis changes.

**Figure 3:**
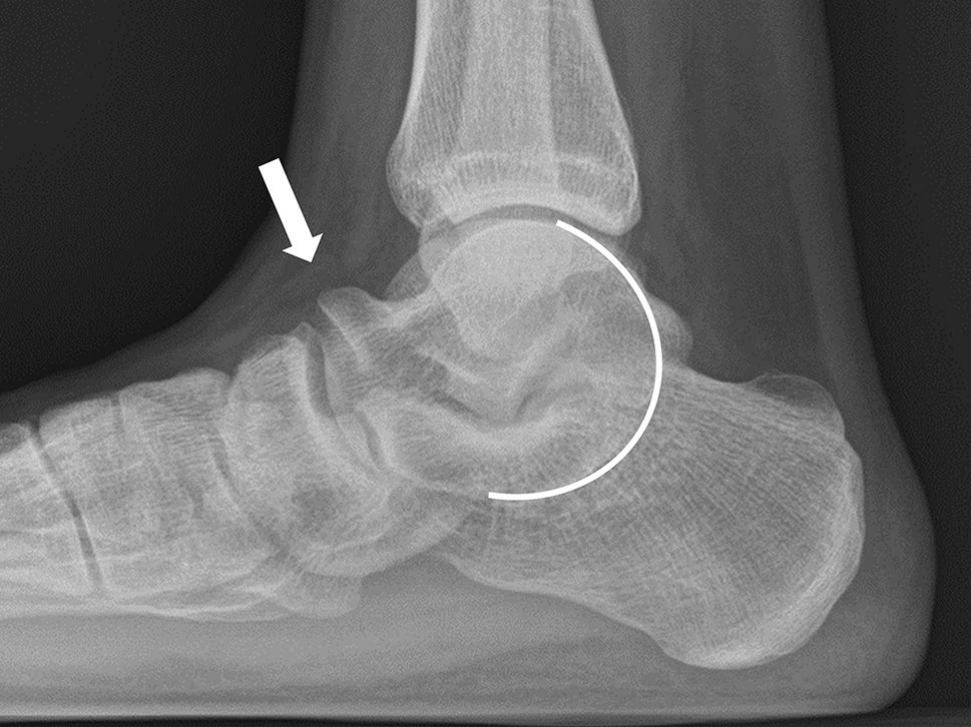
Talar beak sign and C sign on a simple radiograph.

**Figure 4:**
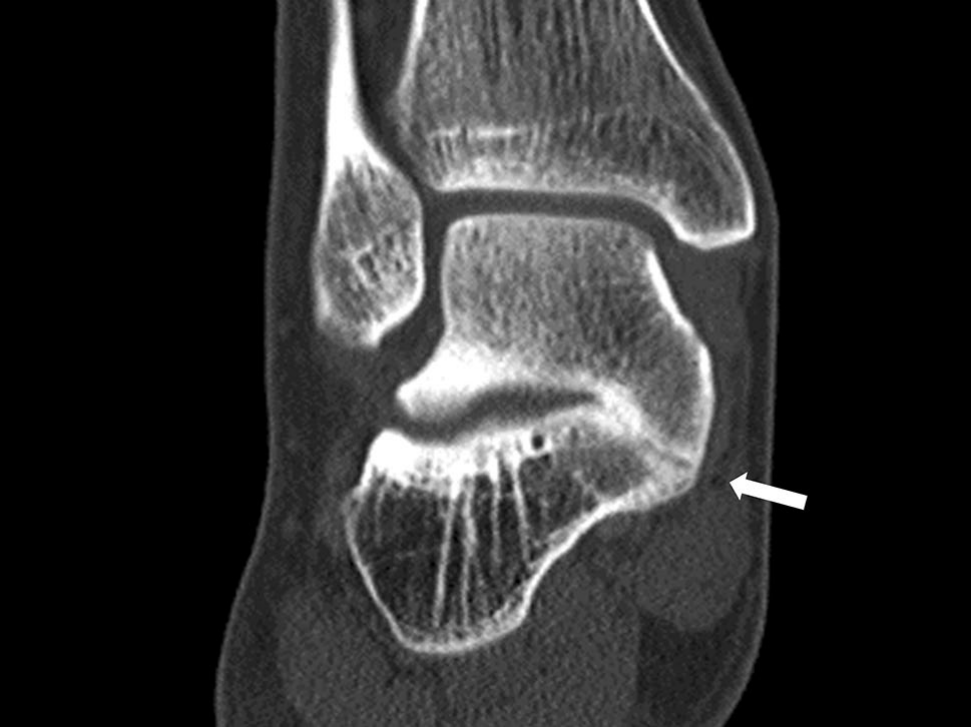
Drunken waiter sign of talocalcaneal coalition.

Anatomical types of tarsal coalition were classified according to the united tarsal bone as TC, CN, naviculocuneiform (NC), talonavicular (TN), and calcaneocuboid (CC) coalition. Anatomical subtypes were further classified according to location of tarsal bone union for TC and NC coalitions. For TC coalition, union was assessed at the anterior, middle, and posterior facet, and for NC coalition, union was assessed at the medial, intermediate, and lateral cuneiform. The middle and posterior facets are apposed and difficult to differentiate. The canalis-tarsi is anatomically located between the middle and posterior facets, and therefore, we carefully differentiated union of the middle and posterior facets (Fig. 1)^30^. In addition, non-osseous TC coalitions may present CT findings similar to degenerative osteoarthritis with abnormal joint space narrowing and minimal marginal reactive osseous changes (Fig. 2)^35^. Although we excluded patients with severe subtalar osteoarthritis before the study, it was important to distinguish between non-osseous TC coalition and mild subtalar osteoarthritis. When there was a subtalar lesion showing the above findings on CT in relatively young patients under 40 years old, it was diagnosed as TC coalition because of the low possibility of degenerative changes. In patients over 40 years old, talar beak sign and C sign were additionally assessed by simple radiography to diagnose TC coalition (Fig. 3)^35,37^. Also, a drunken waiter sign was evaluated on the CT coronal view (Fig. 4)^37^.

Bilateral coalition was defined as coalition in both feet of the same anatomical type. Bilateral occurrence was evaluated for each anatomical type, and its prevalence are presented as percentages of bilateral patients among patients of each anatomical type.

All patients were assessed retrospectively for symptoms related to tarsal coalition. Clinical features were evaluated based on the pathologic condition patients complained of at the time of CT scan and were obtained by chart review. Symptoms associated with tarsal coalition were as follows: repeated ankle sprains and pain, pain at the coalition site, adjacent joint pain due to secondary degeneration, sinus tarsi syndrome, peroneal spasm, and tarsal tunnel syndrome^3,33,38,39^. If there was no other disease causing these symptoms, it was concluded as a symptom caused by tarsal coalition. In addition, pain at the coalition site was judged as a symptom of coalition when there was ill-defined fluid signal intensity in the bone marrow of the adjacent bone. Proportions of symptomatic patients associated with tarsal coalition were identified in main anatomical type of TC and CN coalitions.

We randomly selected 51 CT images of the 51 patients based on the calculation of sample size according to Bonnett’s approximation to assess intra- and inter-observer reliabilities for the diagnosis of tarsal coalition^40^. Diagnoses were performed independently without personal information by two orthopaedic surgeons and repeated 2 weeks later. Reliabilities for diagnosing tarsal coalition were analyzed using Kappa statistics^41^.

The statistical analysis was performed using IBM SPSS version 23 (IBM Corp., Armonk, NY, USA). The chi-square test was used to compare prevalence of tarsal coalition according to gender, and to compare proportions of symptomatic patients associated with tarsal coalition between anatomical types, including CN and TC coalition. The student-t test was used to compare the age according to the presence tarsal coalition. Statistical significance was accepted for *p-*value < 0.05.

## Results

A total of 839 patients were included in this study. Fifty patients had tarsal coalition, an overall prevalence of 6.0%. The proportions of tarsal coalition patients by groups according to age and gender are described in Table 2. Gender (*p* = 0.492) and age (*p* = 0.762) were not significantly associated with the presence of tarsal coalition. The proportion of tarsal coalition patients was the highest in the patient group under 20 years old (11%) (Table 2).

**Table 2:** Proportions of tarsal coalition patients by group according to age and gender.

Variable	Patients with tarsal coalition (%)	Patients without tarsal coalition (%)	*p*-value
Total (n = 839)	50 (6)	789 (94)	
**Gender**			0.492
Male (n = 492)	27 (5.5)	465 (94.5)	
Female (n = 347)	23 (6.6)	324 (93.4)	
**Age (yr)**			0.762
≤ 20 (n = 91)	10 (11)	81 (89)	
21–40 (n = 157)	14 (8.9)	143 (91.1)	
41–60 (n = 308)	12 (3.9)	296 (96.1)	
≥ 61 (n = 283)	14 (4.9)	269 (95.1)	

Values are presented as number (%).

Proportions of anatomical types of tarsal coalition are described in Table 3. Isolated TC coalition was the most common anatomical types with a prevalence of 50.0% (Table 3). Bilateral occurrence of tarsal coalition is described in Table 4. Overall, the bilateral occurrence was 56.5% and TC coalition had the highest bilateral occurrence among anatomical types at 76.0% (Table 4).

**Table 3:** Distribution of anatomical type.

Subtype	Patients with tarsal coalition (n = 50)
Isolated TC coalition	25 (50)
Isolated CN coalition	15 (30)
Isolated NC coalition	6 (12)
CN coalition + TC coalition	2 (4)
TC coalition + TN coalition	1 (2)
TC coalition + CC coalition	1 (2)

Values are presented as number (%).

*TC* talocalcaneal, *CN* calcaneonavicular, *NC* naviculocuneiform, *TN* talonavicular, *CC* calcaneocuboid.

**Table 4:** Bilateral occurrence.

Type	Bilateral occurrence (%)
Total (n = 46)^a^	26 (56.5)
TC coalition (n = 25)	19 (76)
CN coalition (n = 15)	4 (26.7)
NC coalition (n = 6)	3 (50)

Values are presented as number (%).

*TC* talocalcaneal, *CN* calcaneonavicular, *NC* naviculocuneiform.

^a^Multiple tarsal coalition patients are excluded from this list.

Proportions of anatomical subtypes according to union sites of TC and NC coalitions are provided in Table 5. Isolated union of the posterior facet was most common at 43.2% in TC coalitions, and medial cuneiform was most common coalition site in NC coalitions accounting for 77.8% (Table 5). Coalition of multiple cuneiform bones was not observed.

**Table 5:** Detailed subtypes of talocalcaneal and naviculocuneiform coalitions.

Subtype	No. of tarsal coalition
**TC coalition (n = 44)**^**a**^	
Isolated anterior facet	0
Isolated middle facet	7 (15.9)
Isolated posterior facet	19 (43.2)
Anterior facet + middle facet	0
Middle facet + posterior facet	11 (25)
Anterior facet + posterior facet	0
Anterior facet + middle facet + posterior facet	7 (15.9)
**NC coalition (n = 9)**^**a**^	
Medial cuneiform	7 (77.8)
Intermediate cuneiform	2 (22.2)
Lateral cuneiform	0

Values are presented as number (%).

*TC* talocalcaneal, *NC* naviculocuneiform.

^a^Bilateral lesions are counted individually.

The proportion of patients with symptoms related to tarsal coalition in anatomical types including TC and CN coalitions are described in Table 6. TC coalition was significantly more associated with symptoms than CN coalition (*p* = 0.019) (Table 6).

**Table 6:** Proportion of patients with symptoms in main anatomical types.

Subtype	Patient with coalition symptom	Patient without coalition symptom	*p-*value
Total (n = 50)	35 (70)	15 (30)	
Isolated TC coalition (n = 25)	22 (88)	3 (12)	0.019^a^
Isolated CN coalition (n = 15)	7 (46.7)	8 (53.3)	

Values are presented as number (%).

*TC* talocalcaneal, *CN* calcaneonavicular.

^a^The Chi-square test was used to compare proportions of symptomatic patients associated with tarsal coalition between isolated CN and TC coalition.

Intra- and interobserver reliabilities for diagnosing tarsal coalition were 0.938 and 0.916, respectively.

## Discussion

In this study, the overall prevalence of tarsal coalition was 6.0%. A review of articles on the the prevalence of tarsal coalition, including current study, is presented in Table 7^11–13,15,16,31,42^. In order to overcome the limitations of existing literatures, in our study, CT scans were performed in all patients to assess the complex anatomy in detail. In addition, the study was conducted on a relatively large number of patients, including patients without coalition-related symptoms as much as possible. As we expected, this result was appreciably higher than the prevalence of 0.03%–1% from previous studies which assessed tarsal coalition using physical examination or simple radiography^11–13,42^. However, the prevalence was lower than that of recent cadaver and MRI studies^15,16,34^. We believed two factors may have influenced these results. First, considering that the proportion of symptomatic patients was high at 70%, asymptomatic patients may not have been sufficiently included. Second, CT is more vulnerable to the misdiagnosis of non-osseous coalition than MRI. Solomon et al.^34^ reported nine non-osseous coalitions were diagnosed among 100 dissected feet, and only 55% of them were correctly diagnosed by prior CT. Guignand et al.^43^ found that four coalitions (2 cartilaginous and 2 fibrous forms) among 11 CN coalitions diagnosed intraoperatively were not diagnosed by CT, but could be diagnosed by MRI. Therefore, the actual prevalence is expected to be higher. The low prevalence of 1.7% in Kim et al.'s study^31^ using CT and MRI is may be due to the limited study subjects of soldiers treated for ankle sprain or fracture. We found the proportion of tarsal coalition patients in current study was highest at 11% in the patient group under 20 years of age. Since tarsal coalition mostly causes symptoms in adolescence and early adulthood^14,44^, a relatively large number of symptomatic young patients may have visited the hospital. Therefore, it is expected that the probability of receiving a diagnosis by conducting an examination was relatively higher than that of the older group.

**Table 7:** Literature review on the prevalence of tarsal coalition.

Author	No. of subject (feet)	Prevalence (%)	Study subject	Evaluation methods
Harris et al. (1948)^11^	3600	0.03	Army personnel	P/E
Lee et al. (2020)^42^	448	0.4	Healthy and asymptomatic people	XR
Shands et al. (1953)^12^	1232	0.9	Patients of children’s clinic	XR
Vaughan et al. (1953)^13^	2000	1	Army personnel with painful feet	XR
Kim et al. (2020)^31^	4711	1.7	Army personnel who underwent ankle sprain or fracture	CT, MRI
Our study (2022)	1678	6	Patients in hospital	CT
Nalaboff et al. (2008)^16^	667	11.5	Patients in hospital	MRI
Ruhli et al. (2003)^15^	114	13	Cadavers	Cadaver study

Values are presented as number.

*CT* computed tomography, *MRI* magnetic resonance imaging.

In terms of anatomical types, TC coalition was the most common followed by CN coalition. These two types accounted for most of the total tarsal coalition, which concurs with previous studies^14,15^. A review of articles on the the anatomical distribution and bilateral occurrence of tarsal coalition, including current study, is presented in Table 8^14–17,25,29–34,45–47^. Our study was the only one to evaluate not only the anatomical classification and bilaterality of tarsal coalitions, but also the detailed distribution of TC coalitions. In addition, there was no East Asian study that assessed bilateral occurrence except our study. While CN coalition was dominant in many Caucasian studies^14,16,17,46,47^, TC coalition was more common in this study, which is in line with the results from recent East Asian studies^29,31^. These results indicate the anatomical distribution of tarsal coalition is racially dependent. In addition, differences between the clinical presentation of TC and CN coalitions may have contributed reported TC coalition predominance. According Solomon et al.^15,34^, CN coalition appears to be unrelated to arthritic change, which means that limitations of subtalar joint motion are less likely for CN coalition. Since motion restriction causes symptoms of tarsal coalition and secondary arthritis, TC coalition likely accounts for a higher proportion of symptomatic patients than CN coalition. In fact, in this study, the proportion of symptomatic patients was significantly higher for TC coalition. Due to these differences in clinical presentation, TC coalition patients are more likely to visit the hospital and be diagnosed with tarsal coalition. Furthermore, coalition type may have influenced the results of this CT study. According to Nalaboff et al.^16^, TC coalition involves osseous coalition in 33.3% of patients, whereas most of the CN coalition is composed of non-osseous coalition (56% cartilaginous and 44% fibrous union). In the present study, CT was the only imaging modality used, and thus, non-osseous forms of coalition may have been under diagnosed.

**Table 8:** Literature review on the anatomical distribution and bilateral occurrence of tarsal coalition.

Author (year)	Study subject	Overall coalition	CN coalition	TC coalition	Detailed distribution of TC coalition (middle facet/posterior facet/middle and posterior facet)	Bilaterality^a^ (CN coalition/TC coalition)
**Caucasian**						
Taylor et al. (2020)^47^	Paediatric county residents	85	46 (54.1)	30 (35.3)	N/A	N/A
Swiontkowski et al. (1983)^17^	Patients of children’s clinic	57	44 (77.2)	13 (22.8)	N/A	N/A
Khoshbin et al. (2013)^46^	children’s clinic	32	19 (59.4)	13 (40.6)	N/A	N/A
Nalaboff et al. (2008)^16^	Patients in hospital	70	50 (71.4)	18 (25.7)	N/A	N/A
Stormont et al. (1983)^14^	Patients in hospital	60	32 (53.3)	22 (36.7)	N-A	68.4/22.2
Ruhli et al. (2003)^15^	Cadavers	10	8 (80)	2 (20)	N-A	33.3/ 0
Solomon et al. (2003)^33^	Cadavers	9	7 (77.8)	2 (22.2)	N-A	40/ 0
Luhmann et al. (1998)^45^	Patients in hospital	N/A	N/A	25	21 (84)/1 (4)/3 (12)	N-A
Scranton et al. (1987)^25^	Patients in hospital	N/A	N/A	23	13 (56.5)/4 (17.4)/6 (26.1)	N/A
**East Asian**						
Takakura et al. (1991)^28^	Patients in hospital	67	N/A	53 (79.1)	N/A	N/A
Kim et al. (2020)^31^	Army personnel	59	10 (16.9)	31 (62.7)	8 (25.8)/15 (48.4)/5 (16.1)	N/A
Yun et al.(2015)^29^	Patients in hospital	81	6 (7.4)	54 (66.7)	8 (14.8)/28 (51.9)/3 (5.6)	N/A
Lee et al. (2016)^30^	Patients in hospital	N/A	N/A	43	2 (4.7)/29 (67.4)/12 (27.9)	N/A
Wang et al. (2021)^32^	Patients in hospital	N/A	N/A	108	0/29 (26.9)/74 (68.5)	N/A
Our study (2022)	Patients in hospital	46	15 (32.6)	25 (54.3)	7 (15.9)/19 (43.2)/11 (25)	26.7/ 76

Values are presented as number or number (%).

*CN* calcaneonavicular, *TC* talocalcaneal.

^a^Values are presented as percent.

In current study, the bilateral occurrence of tarsal coalition was 56.5% among the 46 patients, which is in line with previous studies^18,48,49^. Regarding anatomical types, TC and CN coalition had bilateral occurrence of 76.0% and 26.7%, respectively. However, several Caucasian studies have reported a higher bilateral occurrence of CN coalition than TC coalition (Table 8)^14–16^. In addition, similar to other East Asian studies, the posterior facet lesion accounted for the highest proportion (43.2%) of TC coalition subtypes, but it was far from the results of the Cacausian studies (Table 8)^25,29–32,45^. These can be explained by racial differences, but additional larger-scale Asian studies are needed to clarify this topic.

The proportions of patients with coalition-related symptoms were compared in TC and CN coalitions. TC coalition (88.0%) accounted for a significantly higher proportion of symptomatic patients than CN coalition (46.7%), which may be the result of less subtalar motion limitation in CN coalition^34^.

Summarizing the characteristics of tarsal coalition in East Asians, tarsal coalition is expected to be much more common than previously reported. TC coalition is the most common anatomical type and has a very high bilateral occurrence. In addition, TC coalition most often invades the posterior facet, and accompanies coalition-related symptoms at a high rate. This suggests that there are relatively more symptomatic tarsal coalition patients than in East Asians than Caucasians. Therefore, it is necessary to consider the possibility of tarsal coalition not only in patients with rigid flat foot deformity but also in East Asian patients who visit the hospital for other foot and ankle symptoms. If tarsal coalition is suspected, it is better to perform CT or MRI together than simple radiograph alone. Also, since patients with coalition-related symptoms are more likely to have TC coalition, bilateral examination is very important for diagnosis.

Our study has several strengths. First, it included a large number (839 subjects) of variously aged patients, and second, bilateral CT was performed on all patients. However, there is a possibility that the diagnosis of non-osseous coalition was insufficient due to non-availability of MRI findings. Performing MRI for research is difficult due to cost burden. Recently, the use of deep learning algorithms in medical imaging is rapidly increasing^50,51^. If a model capable of diagnosing tarsal coalition using imaging findings of CT scans is developed, this problem could be improved. In addition, we depended on the medial records of the hospital to confirm the clinical features at the time of the examination. Therefore, the possibility of insufficient evaluation on other pathological conditions that may cause symptoms cannot be excluded.

## Conclusion

The prevalence of tarsal coalition in our study was found to be 6%, which is higher than previously reported. TC coalition was the most common coalition type and usually involved the posterior facet, which contrasts with that reported for Caucasians. In addition, talocalcaneal coalition was associated with a high prevalence of bilateral involvement and a high rate of coalition-related symptoms. The possibility of tarsal coalition in foot and ankle patients in East Asians should always be considered. When tarsal coalition is suspected, bilateral examination is essential for diagnosis.

## Data Availability

Data and materials used and analysed during the current study are available from the corresponding author on reasonable request.
